# Crystal structure of a looped-chain Co^II^ coordination polymer: *catena*-poly[[bis­(nitrato-κ*O*)cobalt(II)]bis­[μ-bis­(pyridin-3-ylmeth­yl)sulfane-κ^2^ *N*:*N*′]]

**DOI:** 10.1107/S2056989017014980

**Published:** 2017-10-20

**Authors:** Suk-Hee Moon, Joobeom Seo, Ki-Min Park

**Affiliations:** a Department of Food and Nutrition, Kyungnam College of Information and Technology, Busan 47011, Republic of Korea; b Mineral Resources Research Division, Korea Institute of Geoscience and Mineral Resources (KIGAM), Daejeon 34132, Republic of Korea; c Research institute of Natural Science, Gyeongsang National University, Jinju 52828, Republic of Korea

**Keywords:** crystal structure, cobalt(II), dipyridyl ligand, looped chain, hydrogen bonding, π–π inter­actions

## Abstract

The asymmetric unit of the title compound, [Co(NO_3_)_2_(C_12_H_12_N_2_S)_2_]_*n*_, contains a bis­(pyridin-3-ylmeth­yl)sulfane (*L*) ligand, an NO_3_^−^ anion and half a Co^II^ cation, which lies on an inversion centre. The Co^II^ cation is six-coordinated, being bound to four pyridine N atoms from four symmetry-related *L* ligands. The remaining coordination sites are occupied by two O atoms from two symmetry-related nitrate anions in a monodentate manner. Thus, the Co^II^ centre adopts a distorted octa­hedral geometry. Two symmetry-related *L* ligands are connected by two symmetry-related Co^II^ cations, forming a 20-membered cyclic dimer, in which the Co^II^ atoms are separated by 10.2922 (7) Å. The cyclic dimers are connected to each other by sharing Co^II^ atoms, giving rise to the formation of an infinite looped chain propagating along the [101] direction. Inter­molecular C—H⋯π (H⋯ring centroid = 2.89 Å) inter­actions between one pair of corresponding *L* ligands and C—H⋯O hydrogen bonds between the *L* ligands and the nitrate anions occur in the looped chain. In the crystal, adjacent looped chains are connected by inter­molecular π–π stacking inter­actions [centroid-to-centroid distance = 3.8859 (14) Å] and C—H⋯π hydrogen bonds (H⋯ring centroid = 2.65 Å), leading to the formation of layers parallel to (101). These layers are further connected through C—H⋯O hydrogen bonds between the layers, resulting in the formation of a three-dimensional supra­molecular architecture.

## Abstract 2

The reaction of cobalt(II) nitrate with bis­(pyridin-3-ylmeth­yl)sulfane ligand, afforded a one-dimensional looped polymeric chain. The Co^II^ cation displays a distorted octa­hedral geometry coordinated by four pyridine N atoms from four individual ligands and two O atoms from two monodentate nitrate anions. Two symmetry-related ligands are connected by two symmetry-related Co^II^ cations, forming a 20-membered cyclic dimer. These cyclic dimers are connected to each other by sharing Co^II^ atoms, forming a looped chain. In the crystal, adjacent looped chains are connected by inter­molecular π–π stacking inter­actions and C—H⋯π and C—H⋯O hydrogen bonds, resulting in the formation of a three-dimensional supra­molecular architecture.

## Chemical context   

Over the last two decades, numerous one-dimensional coord­ination polymers have been developed, not only because of their fascinating architectures but also their potential applications as functional materials (Furukawa *et al.*, 2014 ▸; Silva *et al.*, 2015 ▸). In this area of research, dipyridyl-type mol­ecules as organic building blocks have been widely used to construct diverse one-dimensional self-assembled coordination polymers with intriguing structural topologies (Leong & Vittal, 2011 ▸; Wang *et al.*, 2012 ▸). Our group has also developed several one-dimensional coordination polymers with fascinating topologies such as zigzag (Lee *et al.*, 2013 ▸; Moon *et al.*, 2016 ▸), helical (Moon *et al.*, 2014 ▸, 2015 ▸), double helical (Lee *et al.*, 2015 ▸), looped chain (Ju *et al.*, 2014 ▸) and ribbon-type double-stranded (Moon *et al.*, 2017 ▸; Park *et al.*, 2010 ▸) structures using dipyridyl-type ligands. In an extension of our research, the title compound was prepared by the reaction of cobalt(II) nitrate with bis­(pyridin-3-ylmeth­yl)sulfane (*L*) as a flexible dipyridyl-type ligand, synthesized using a literature procedure (Park *et al.*, 2010 ▸; Lee *et al.*, 2012 ▸). Herein, we report the crystal structure of the title compound, which adopts a one-dimensional looped-chain structure.
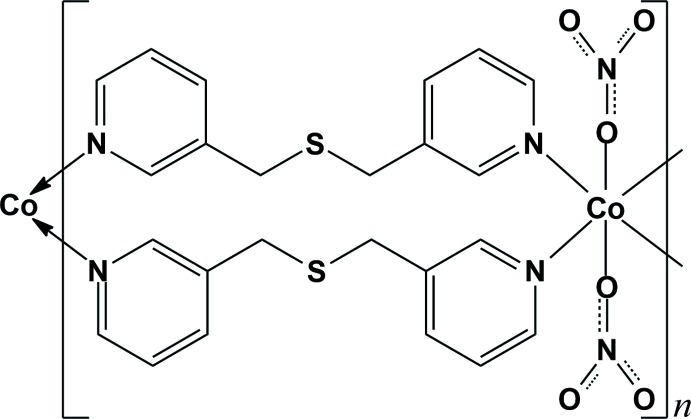


## Structural commentary   

**Figure 1:**
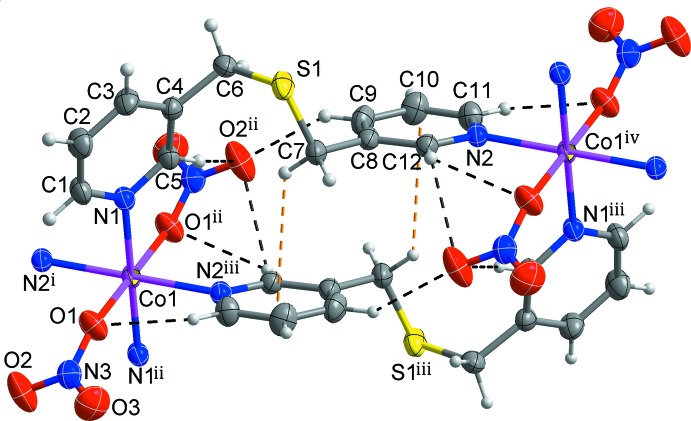
View of the cyclic dimer structure of the title compound, showing the geometry around Co^II^ centre and the atom-numbering scheme. Displacement ellipsoids are drawn at the 50% probability level. Inter­molecular C—H⋯π and C—H⋯O hydrogen bonds are represented by yellow and black dashed lines, respectively [symmetry codes: (i) *x* + 1, *y*, *z* + 1; (ii) −*x* + 1, −*y* + 1, −*z* + 1; (iii) −*x*, −*y* + 1, −*z*; (iv) *x* − 1, *y*, *z* − 1].

As illustrated in Fig. 1 ▸, the asymmetric unit of the title compound consists of one Co^II^ cation located on an inversion centre, one (pyridin-3-ylmeth­yl)sulfane ligand, *L*, and one NO_3_^−^ anion. The Co^II^ cation is coordinated by four pyridine N atoms from four symmetry-related *L* ligands. In addition, the Co^II^ cation binds to two O atoms of two symmetry-related monodentate nitrate anions, forming a distorted octa­hedral CoN_4_O_2_ coordination. Selected bond lengths and angles around the Co1 atom are listed in Table 1 ▸. The N1- and N2-pyridine rings coordinated to the Co^II^ centre are tilted by 70.75 (7)° with respect to each other (Fig. 1 ▸).

Two symmetry-related *L* ligands bridge two Co^II^ atoms, resulting in the formation of a 20-membered cyclic dimer with a Co⋯Co separation of 10.2922 (7) Å. The cyclic dimers are connected by sharing Co^II^ atoms, leading to the formation of an infinite looped chain propagating along the [101] direction. An inter­molecular C7—H7*B*⋯ *Cg*2^i^ inter­actions [H⋯π = 2.89 Å; Table 2 ▸; yellow dashed lines in Fig. 1 ▸; *Cg*2 is the centroid of atoms N2/C8–C12; symmetry code: (i) −*x*, −*y* + 1, −*z*] between one pair of corresponding *L* ligands and several C—H⋯O hydrogen bonds between the *L* ligands and the NO_3_^−^ anions (Table 2 ▸; black dashed lines in Fig. 1 ▸) contribute to the stabilization of the looped chain.

## Supra­molecular features   

**Figure 2:**
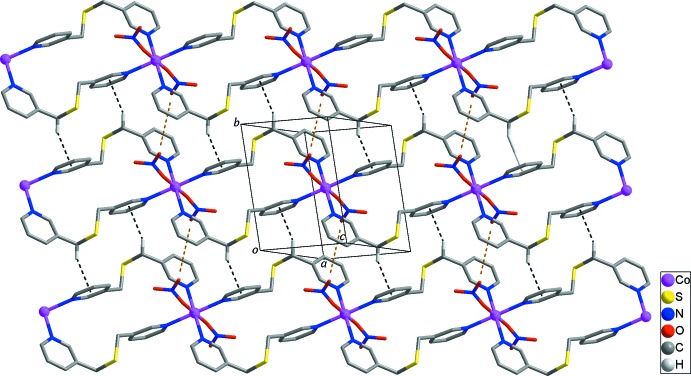
The supra­molecular layer formed by inter­molecular π–π stacking inter­actions (yellow dashed lines) and C—H⋯π hydrogen bonds (black dashed lines) between the looped chains. H atoms not involved in inter­molecular inter­actions have been omitted for clarity.

**Figure 3:**
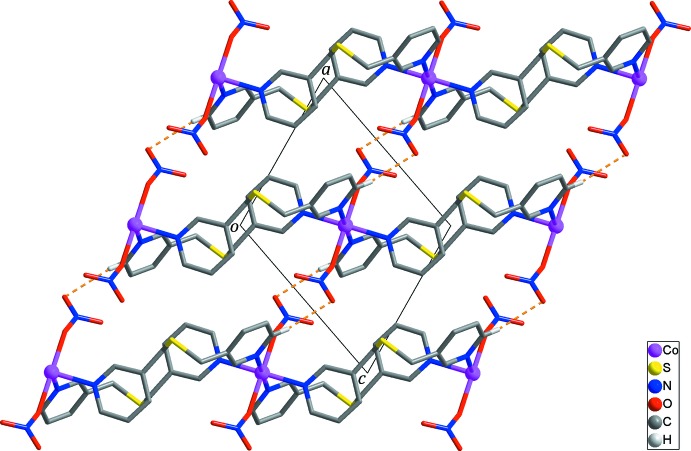
The three-dimensional supra­molecular network formed *via* inter­molecular C—H⋯O hydrogen bonds (yellow dashed lines) between the layers. H atoms not involved in inter­molecular inter­actions have been omitted for clarity.

Adjacent looped chains in the structure are connected by inter­molecular π–π stacking inter­actions between the N1-pyridine rings [*Cg*1⋯*Cg*1^ii^ = 3.8859 (14) Å; yellow dashed lines in Fig. 2 ▸; *Cg*1 is the centroid of atoms N1/C1–C5; symmetry code: (ii) −*x* + 1, −*y* + 2, −*z* + 1] together with inter­molecular C6—H6*A*⋯*Cg*2^iii^ hydrogen bonds [H⋯π = 2.65 Å; Table 2 ▸; black dashed lines in Fig. 2 ▸; symmetry code: (iii) −*x*, −*y* + 2, −*z*], generating layers parallel to (101). Neighboring layers are packed by C1—H1⋯O3^iv^ hydrogen bonds [H⋯O = 2.60 Å; Table 2 ▸; yellow dashed lines in Fig. 3 ▸; symmetry code: (iv) −*x* + 2, −*y* + 1, −*z* + 1] between pyridine H atoms and nitro­gen O atoms, resulting in the formation of a three-dimensional supra­molecular architecture.

## Synthesis and crystallization   

The *L* ligand was synthesized according to a literature method (Park *et al.*, 2010 ▸; Lee *et al.*, 2012 ▸). Crystals of the title compound were grown by slow evaporation of a methanol/H_2_O (2:1) solution of the *L* ligand with Co(NO_3_)_2_·6H_2_O in a 2:1 molar ratio.

## Refinement   

Crystal data, data collection and structure refinement details are summarized in Table 3 ▸. All H atoms were positioned geometrically and refined as riding: C—H = 0.93 Å for C*sp* ^2^—H and 0.97 Å for methyl­ene C—H with *U*_iso_(H) = 1.2*U*_eq_(C).

## Supplementary Material

Crystal structure: contains datablock(s) I, New_Global_Publ_Block. DOI: 10.1107/S2056989017014980/hg5499sup1.cif

Structure factors: contains datablock(s) I. DOI: 10.1107/S2056989017014980/hg5499Isup2.hkl

CCDC reference: 1580230

Additional supporting information:  crystallographic information; 3D view; checkCIF report

## Figures and Tables

**Table 1:** Selected geometric parameters (Å, °)

Co1—O1	2.1414 (16)	Co1—N2^i^	2.1907 (18)
Co1—N1	2.1571 (17)		
			
O1^ii^—Co1—O1	180.0	N1—Co1—N2^i^	92.32 (6)
O1—Co1—N1	85.34 (7)	O1—Co1—N2^iii^	88.21 (7)
O1—Co1—N1^ii^	94.66 (7)	N1—Co1—N2^iii^	87.68 (6)
N1—Co1—N1^ii^	180.0	N2^i^—Co1—N2^iii^	180.0
O1—Co1—N2^i^	91.79 (7)		

Symmetry codes: (i) 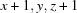; (ii) 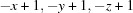; (iii) 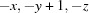.

**Table 2:** Hydrogen-bond geometry (Å, °) *Cg*2 is the centroid of the N2/C8–C12 ring.

*D*—H⋯*A*	*D*—H	H⋯*A*	*D*⋯*A*	*D*—H⋯*A*
C1—H1⋯O3^iv^	0.93	2.60	3.466 (3)	155
C5—H5⋯O2^ii^	0.93	2.30	3.171 (3)	157
C9—H9⋯O2^ii^	0.93	2.54	3.373 (3)	149
C11—H11⋯O1^iii^	0.93	2.43	3.032 (3)	122
C12—H12⋯O1^v^	0.93	2.53	3.134 (3)	123
C12—H12⋯O2^v^	0.93	2.59	3.219 (3)	125
C6—H6*A*⋯*Cg*2^vi^	0.97	2.65	3.546 (3)	154
C7—H7*B*⋯*Cg*2^iii^	0.97	2.89	3.565 (3)	127

Symmetry codes: (ii) 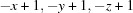; (iii) 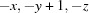; (iv) 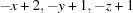; (v) 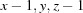; (vi) 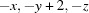.

**Table 3:** Experimental details

Crystal data	
Chemical formula	[Co(NO_3_)_2_(C_12_H_12_N_2_S)_2_]
*M*_r_	615.54
Crystal system, space group	Triclinic, *P* 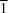
Temperature (K)	298
*a*, *b*, *c* (Å)	8.1620 (7), 8.8158 (8), 9.5078 (8)
α, β, γ (°)	98.531 (2), 109.218 (2), 92.062 (2)
*V* (Å^3^)	636.22 (10)
*Z*	1
Radiation type	Mo *K*α
μ (mm^−1^)	0.89
Crystal size (mm)	0.45 × 0.30 × 0.15

Data collection	
Diffractometer	Bruker APEXII CCD area detector
Absorption correction	Multi-scan (*SADABS*; Bruker, 2014 ▸)
*T*_min_, *T*_max_	0.634, 0.896
No. of measured, independent and observed [*I* > 2σ(*I*)] reflections	3659, 2456, 2082
*R*_int_	0.040
(sin θ/λ)_max_ (Å^−1^)	0.617

Refinement	
*R*[*F* ^2^ > 2σ(*F* ^2^)], *wR*(*F* ^2^), *S*	0.033, 0.088, 1.04
No. of reflections	2456
No. of parameters	178
H-atom treatment	H-atom parameters constrained
Δρ_max_, Δρ_min_ (e Å^−3^)	0.33, −0.37

Computer programs: *APEX2* and *SAINT* (Bruker, 2014 ▸), *SHELXS97* and *SHELXTL* (Sheldrick, 2008 ▸), *SHELXL2014/7* (Sheldrick, 2015 ▸), *DIAMOND* (Brandenburg, 2010 ▸) and *publCIF* (Westrip, 2010 ▸).

## supplementary crystallographic information

### Crystal data

**Table:**

[Co(NO_3_)_2_(C_12_H_12_N_2_S)_2_]	*Z* = 1
*M**_r_* = 615.54	*F*(000) = 317
Triclinic, *P*1	*D*_x_ = 1.607 Mg m^−^^3^
*a* = 8.1620 (7) Å	Mo *K*α radiation, λ = 0.71073 Å
*b* = 8.8158 (8) Å	Cell parameters from 3659 reflections
*c* = 9.5078 (8) Å	θ = 2.3–26.0°
α = 98.531 (2)°	µ = 0.89 mm^−^^1^
β = 109.218 (2)°	*T* = 298 K
γ = 92.062 (2)°	Plate, violet
*V* = 636.22 (10) Å^3^	0.45 × 0.30 × 0.15 mm

### Data collection

**Table:**

Bruker APEXII CCD area detector diffractometer	2082 reflections with *I* > 2σ(*I*)
φ and ω scans	*R*_int_ = 0.040
Absorption correction: multi-scan (SADABS; Bruker, 2014)	θ_max_ = 26.0°, θ_min_ = 2.3°
*T*_min_ = 0.634, *T*_max_ = 0.896	*h* = −7→10
3659 measured reflections	*k* = −10→10
2456 independent reflections	*l* = −11→7

### Refinement

**Table:**

Refinement on *F*^2^	0 restraints
Least-squares matrix: full	Hydrogen site location: inferred from neighbouring sites
*R*[*F*^2^ > 2σ(*F*^2^)] = 0.033	H-atom parameters constrained
*wR*(*F*^2^) = 0.088	*w* = 1/[σ^2^(*F*_o_^2^) + (0.0488*P*)^2^ + 0.1617*P*] where *P* = (*F*_o_^2^ + 2*F*_c_^2^)/3
*S* = 1.04	(Δ/σ)_max_ < 0.001
2456 reflections	Δρ_max_ = 0.33 e Å^−^^3^
178 parameters	Δρ_min_ = −0.37 e Å^−^^3^

### Special details

**Table:**

Geometry. All esds (except the esd in the dihedral angle between two l.s. planes) are estimated using the full covariance matrix. The cell esds are taken into account individually in the estimation of esds in distances, angles and torsion angles; correlations between esds in cell parameters are only used when they are defined by crystal symmetry. An approximate (isotropic) treatment of cell esds is used for estimating esds involving l.s. planes.

### Fractional atomic coordinates and isotropic or equivalent isotropic displacement parameters (Å^2^)

**Table:**

	*x*	*y*	*z*	*U*_iso_*/*U*_eq_	
Co1	0.5000	0.5000	0.5000	0.02620 (13)	
S1	0.20509 (7)	0.89446 (7)	−0.01804 (7)	0.03834 (17)	
N1	0.5427 (2)	0.7131 (2)	0.42595 (19)	0.0294 (4)	
N2	−0.3498 (2)	0.5946 (2)	−0.2639 (2)	0.0294 (4)	
N3	0.8472 (2)	0.3452 (2)	0.4702 (2)	0.0353 (4)	
O1	0.7307 (2)	0.4382 (2)	0.4536 (2)	0.0459 (4)	
O2	0.9392 (2)	0.3314 (3)	0.5987 (2)	0.0623 (6)	
O3	0.8698 (3)	0.2729 (2)	0.3594 (2)	0.0600 (5)	
C1	0.7012 (3)	0.7803 (3)	0.4459 (3)	0.0359 (5)	
H1	0.7992	0.7378	0.5020	0.043*	
C2	0.7239 (3)	0.9093 (3)	0.3867 (3)	0.0419 (6)	
H2	0.8356	0.9533	0.4041	0.050*	
C3	0.5807 (3)	0.9731 (3)	0.3014 (3)	0.0377 (5)	
H3	0.5945	1.0595	0.2595	0.045*	
C4	0.4158 (3)	0.9065 (2)	0.2792 (2)	0.0293 (4)	
C5	0.4044 (3)	0.7788 (2)	0.3446 (2)	0.0288 (4)	
H5	0.2939	0.7352	0.3316	0.035*	
C6	0.2546 (3)	0.9672 (3)	0.1822 (3)	0.0345 (5)	
H6A	0.2707	1.0788	0.1994	0.041*	
H6B	0.1566	0.9379	0.2119	0.041*	
C7	0.1232 (3)	0.6961 (3)	−0.0308 (3)	0.0345 (5)	
H7A	0.1544	0.6312	−0.1080	0.041*	
H7B	0.1802	0.6624	0.0648	0.041*	
C8	−0.0710 (3)	0.6735 (2)	−0.0675 (2)	0.0298 (5)	
C9	−0.1533 (3)	0.7008 (3)	0.0387 (3)	0.0390 (5)	
H9	−0.0889	0.7344	0.1402	0.047*	
C10	−0.3334 (3)	0.6773 (3)	−0.0089 (3)	0.0425 (6)	
H10	−0.3915	0.6973	0.0603	0.051*	
C11	−0.4259 (3)	0.6242 (3)	−0.1590 (3)	0.0369 (5)	
H11	−0.5466	0.6082	−0.1889	0.044*	
C12	−0.1752 (3)	0.6202 (2)	−0.2167 (2)	0.0292 (4)	
H12	−0.1205	0.6011	−0.2885	0.035*	

### Atomic displacement parameters (Å^2^)

**Table:**

	*U*^11^	*U*^22^	*U*^33^	*U*^12^	*U*^13^	*U*^23^
Co1	0.0226 (2)	0.0291 (2)	0.0237 (2)	0.00094 (15)	0.00236 (16)	0.00769 (16)
S1	0.0360 (3)	0.0407 (3)	0.0324 (3)	−0.0055 (2)	0.0012 (2)	0.0141 (2)
N1	0.0260 (9)	0.0313 (9)	0.0286 (9)	0.0012 (7)	0.0049 (7)	0.0082 (7)
N2	0.0280 (9)	0.0285 (9)	0.0279 (9)	−0.0005 (7)	0.0039 (7)	0.0067 (7)
N3	0.0288 (10)	0.0424 (11)	0.0357 (11)	−0.0022 (8)	0.0122 (8)	0.0074 (9)
O1	0.0323 (9)	0.0589 (11)	0.0496 (10)	0.0161 (8)	0.0135 (8)	0.0168 (9)
O2	0.0407 (10)	0.1040 (17)	0.0441 (11)	0.0230 (11)	0.0094 (9)	0.0258 (11)
O3	0.0686 (13)	0.0642 (13)	0.0510 (12)	0.0086 (10)	0.0314 (10)	−0.0046 (10)
C1	0.0243 (11)	0.0422 (13)	0.0367 (12)	0.0025 (9)	0.0023 (9)	0.0114 (10)
C2	0.0292 (12)	0.0469 (14)	0.0478 (14)	−0.0051 (10)	0.0093 (11)	0.0133 (11)
C3	0.0368 (12)	0.0334 (12)	0.0398 (13)	−0.0066 (9)	0.0077 (10)	0.0113 (10)
C4	0.0308 (11)	0.0282 (10)	0.0247 (10)	0.0002 (8)	0.0044 (9)	0.0040 (8)
C5	0.0238 (10)	0.0331 (11)	0.0274 (10)	−0.0003 (8)	0.0056 (8)	0.0067 (9)
C6	0.0337 (12)	0.0285 (11)	0.0370 (12)	0.0035 (9)	0.0042 (10)	0.0100 (9)
C7	0.0305 (11)	0.0330 (11)	0.0311 (11)	0.0001 (9)	−0.0008 (9)	0.0047 (9)
C8	0.0315 (11)	0.0254 (10)	0.0276 (11)	−0.0008 (8)	0.0027 (9)	0.0071 (8)
C9	0.0440 (13)	0.0419 (13)	0.0249 (11)	−0.0050 (10)	0.0051 (10)	0.0037 (9)
C10	0.0441 (14)	0.0520 (15)	0.0334 (12)	−0.0032 (11)	0.0181 (11)	0.0039 (11)
C11	0.0321 (12)	0.0444 (13)	0.0347 (12)	−0.0005 (10)	0.0117 (10)	0.0083 (10)
C12	0.0294 (11)	0.0279 (10)	0.0282 (11)	0.0004 (8)	0.0072 (9)	0.0052 (8)

### Geometric parameters (Å, º)

**Table:**

Co1—O1^i^	2.1414 (16)	C2—H2	0.9300
Co1—O1	2.1414 (16)	C3—C4	1.386 (3)
Co1—N1	2.1571 (17)	C3—H3	0.9300
Co1—N1^i^	2.1571 (17)	C4—C5	1.378 (3)
Co1—N2^ii^	2.1907 (18)	C4—C6	1.505 (3)
Co1—N2^iii^	2.1907 (18)	C5—H5	0.9300
S1—C6	1.820 (2)	C6—H6A	0.9700
S1—C7	1.822 (2)	C6—H6B	0.9700
N1—C1	1.346 (3)	C7—C8	1.505 (3)
N1—C5	1.346 (3)	C7—H7A	0.9700
N2—C11	1.338 (3)	C7—H7B	0.9700
N2—C12	1.345 (3)	C8—C9	1.384 (3)
N2—Co1^iv^	2.1907 (17)	C8—C12	1.392 (3)
N3—O3	1.219 (3)	C9—C10	1.386 (3)
N3—O2	1.232 (3)	C9—H9	0.9300
N3—O1	1.264 (2)	C10—C11	1.376 (3)
C1—C2	1.374 (3)	C10—H10	0.9300
C1—H1	0.9300	C11—H11	0.9300
C2—C3	1.378 (3)	C12—H12	0.9300
			
O1^i^—Co1—O1	180.0	C4—C3—H3	120.5
O1^i^—Co1—N1	94.66 (7)	C5—C4—C3	117.63 (19)
O1—Co1—N1	85.34 (7)	C5—C4—C6	120.95 (19)
O1^i^—Co1—N1^i^	85.34 (7)	C3—C4—C6	121.38 (19)
O1—Co1—N1^i^	94.66 (7)	N1—C5—C4	124.37 (19)
N1—Co1—N1^i^	180.0	N1—C5—H5	117.8
O1^i^—Co1—N2^ii^	88.21 (7)	C4—C5—H5	117.8
O1—Co1—N2^ii^	91.79 (7)	C4—C6—S1	112.23 (16)
N1—Co1—N2^ii^	92.32 (6)	C4—C6—H6A	109.2
N1^i^—Co1—N2^ii^	87.68 (6)	S1—C6—H6A	109.2
O1^i^—Co1—N2^iii^	91.79 (7)	C4—C6—H6B	109.2
O1—Co1—N2^iii^	88.21 (7)	S1—C6—H6B	109.2
N1—Co1—N2^iii^	87.68 (6)	H6A—C6—H6B	107.9
N1^i^—Co1—N2^iii^	92.32 (6)	C8—C7—S1	113.88 (15)
N2^ii^—Co1—N2^iii^	180.0	C8—C7—H7A	108.8
C6—S1—C7	101.13 (11)	S1—C7—H7A	108.8
C1—N1—C5	116.71 (18)	C8—C7—H7B	108.8
C1—N1—Co1	123.98 (14)	S1—C7—H7B	108.8
C5—N1—Co1	119.10 (13)	H7A—C7—H7B	107.7
C11—N2—C12	116.94 (19)	C9—C8—C12	117.5 (2)
C11—N2—Co1^iv^	121.87 (15)	C9—C8—C7	124.0 (2)
C12—N2—Co1^iv^	121.15 (14)	C12—C8—C7	118.5 (2)
O3—N3—O2	120.9 (2)	C8—C9—C10	118.8 (2)
O3—N3—O1	119.8 (2)	C8—C9—H9	120.6
O2—N3—O1	119.3 (2)	C10—C9—H9	120.6
N3—O1—Co1	145.89 (15)	C11—C10—C9	119.7 (2)
N1—C1—C2	122.6 (2)	C11—C10—H10	120.2
N1—C1—H1	118.7	C9—C10—H10	120.2
C2—C1—H1	118.7	N2—C11—C10	122.9 (2)
C1—C2—C3	119.8 (2)	N2—C11—H11	118.6
C1—C2—H2	120.1	C10—C11—H11	118.6
C3—C2—H2	120.1	N2—C12—C8	124.2 (2)
C2—C3—C4	118.9 (2)	N2—C12—H12	117.9
C2—C3—H3	120.5	C8—C12—H12	117.9
			
O3—N3—O1—Co1	122.6 (3)	C7—S1—C6—C4	73.67 (17)
O2—N3—O1—Co1	−59.0 (4)	C6—S1—C7—C8	91.15 (17)
C5—N1—C1—C2	−0.2 (3)	S1—C7—C8—C9	−82.8 (2)
Co1—N1—C1—C2	174.55 (18)	S1—C7—C8—C12	98.0 (2)
N1—C1—C2—C3	−0.9 (4)	C12—C8—C9—C10	−1.4 (3)
C1—C2—C3—C4	1.0 (4)	C7—C8—C9—C10	179.4 (2)
C2—C3—C4—C5	0.1 (3)	C8—C9—C10—C11	1.5 (4)
C2—C3—C4—C6	−177.6 (2)	C12—N2—C11—C10	−0.5 (3)
C1—N1—C5—C4	1.4 (3)	Co1^iv^—N2—C11—C10	177.10 (18)
Co1—N1—C5—C4	−173.66 (16)	C9—C10—C11—N2	−0.6 (4)
C3—C4—C5—N1	−1.3 (3)	C11—N2—C12—C8	0.6 (3)
C6—C4—C5—N1	176.39 (19)	Co1^iv^—N2—C12—C8	−177.01 (15)
C5—C4—C6—S1	−95.4 (2)	C9—C8—C12—N2	0.4 (3)
C3—C4—C6—S1	82.2 (2)	C7—C8—C12—N2	179.61 (19)

Symmetry codes: (i) −*x*+1, −*y*+1, −*z*+1; (ii) *x*+1, *y*, *z*+1; (iii) −*x*, −*y*+1, −*z*; (iv) *x*−1, *y*, *z*−1.

### Hydrogen-bond geometry (Å, º)

*Cg*2 is the centroid of the N2/C8–C12 ring.

**Table:**

*D*—H···*A*	*D*—H	H···*A*	*D*···*A*	*D*—H···*A*
C1—H1···O3^v^	0.93	2.60	3.466 (3)	155
C5—H5···O2^i^	0.93	2.30	3.171 (3)	157
C9—H9···O2^i^	0.93	2.54	3.373 (3)	149
C11—H11···O1^iii^	0.93	2.43	3.032 (3)	122
C12—H12···O1^iv^	0.93	2.53	3.134 (3)	123
C12—H12···O2^iv^	0.93	2.59	3.219 (3)	125
C6—H6*A*···*Cg*2^vi^	0.97	2.65	3.546 (3)	154
C7—H7*B*···*Cg*2^iii^	0.97	2.89	3.565 (3)	127

Symmetry codes: (i) −*x*+1, −*y*+1, −*z*+1; (iii) −*x*, −*y*+1, −*z*; (iv) *x*−1, *y*, *z*−1; (v) −*x*+2, −*y*+1, −*z*+1; (vi) −*x*, −*y*+2, −*z*.
